# The Microdamage and Expression of Sclerostin in Peri-implant Bone under One-time Shock Force Generated by Impact

**DOI:** 10.1038/s41598-017-06867-9

**Published:** 2017-07-26

**Authors:** Xiaoou Diao, Zhirui Li, Baili An, Haitao Xin, Yulu Wu, Kai Li, Fan Feng, Chenyun Dou

**Affiliations:** 1 State Key Laboratory of Military Stomatology, Department of Prosthodontics, School of Stomatology, The Fourth Military Medical University, Xi’an, 710032 China; 2 National Clinical Research Center for Oral Diseases, Department of Prosthodontics, School of Stomatology, The Fourth Military Medical University, Xi’an, 710032 China; 3 Shaanxi Key Laboratory of Oral Diseases, Department of Prosthodontics, School of Stomatology, The Fourth Military Medical University, Xi’an, 710032 China

## Abstract

Osseointegration is the key to implant stability and occlusal support. Biomechanical response and remodeling of peri-implant bone occurs under impact loading. Sclerostin participates in bone formation and resorption through Wnt and RANKL pathways. However the mechanism of microdamage and expression of sclerostin in peri-implant bone under impact load is still unclear. In present study, specific impact forces were applied to the implants with favorable osseointegration in rabbits. The microdamage of peri-implant bone and the expression of sclerostin, β-catenin and RANKL during the process of bone damage and remodeling were investigated by micro-CT, histology, immunofluorescence and RT-qPCR analysis. Interface separation and trabecular fracture were found histologically, which were consistent with micro-CT analyses. Throughout remodeling, bone resorption was observed during the first 14 days after impact, and osseointegration and normal trabecular structure were found by 28 d. The expression of sclerostin and RANKL increased after impact and reached a maximum by 14 d, then decreased gradually to normal levels by 28 d. And β-catenin expression was opposite. Results indicated that sclerostin may involve in the peri-implant bone damage caused by impact and remodeling through Wnt/β-catenin and RANKL/RANK pathways. It will provide a new insight in the diagnosis and treatment for patients suffering impact.

## Introduction

Implant dentures are supported by dental implants, which are inserted into the alveolar bone and connect directly to the bone without intervening soft tissue, following osseointegration^1–3^. Osseointegration is very important for an implant to maintain its stability and provide occlusal support^4^. Occlusal force is transmitted from the implant to the alveolar bone by functional loading during mastication. The biomechanical response of the bone to proper occlusal force determines the osseointegration of the implant and bone remodeling after implantation^5–8^. A failed osseointegration would decrease the stability of the implant and cause the failure of the restoration.

Implant dentures may suffer from impact, particularly during activities such as sports and physical training. Impact is a complex phenomenon that occurs when two or more bodies undergo a collision^9^. Characteristics of impact are very brief duration, high force levels reached, rapid dissipation of energy and large accelerations and decelerations present^9^. When the pulse duration of the impact load is in the range of microseconds, the force is transmitted and reflected, usually in the form of stress waves, through the composite structure consisting of the implant, the interface and the alveolar bone, in which each has the different impedance. If the dental implant and alveolar bone are unable to buffer the impact through deformation of their structure, the osseointegration at the implant-bone interface and the bone microstructure around the implant would be damaged. However, the mechanism of trauma is still unclear.

The bone is a functional remodeling biomaterial. Bone remodeling is usually defined as a process where bone gradually alters its morphology when mechanical signals are sensed and conducted by osteocytes through the lacunar-canalicular system to adapt to the biomechanical environment^10–16^. Bone remodeling includes two opposing processes: resorption and deposition. Sclerostin secreted by osteocytes is a glycoprotein encoded by the SOST gene^17, 18^. Several studies have reported that sclerostin plays important roles in the anabolic response of bone to mechanical stimulation through the Wnt/β-catenin pathway and the catabolic response of bone to mechanical stimulation through the RANK/RANKL pathway^19–22^. However, the expression of sclerostin in peri-implant bone following impact is still unclear, and the correlation between the expression of sclerostin and bone remodeling needs to be studied.

In order to address these questions, we established an animal model with implants under impact load. The characteristics of the microdamage and the expression of sclerostin, β-catenin and RANKL were investigated to explore the mechanism of impact damage and the subsequent repair of bone around the implant. These results could help in the evaluation of alveolar bone trauma around implants and provide guidance for the management of dental implants following impact.

## Materials and Methods

### Animal Studies

#### Animal Preparation

Thirty 20- to 22-week-old female New Zealand rabbits weighting approximately 3.5–4.0 kg were purchased from the animal centre of the Fourth Military Medical University (Shaanxi, China) and housed for 1 month. The animals were allowed access to water and pelleted commercial diet ad libitum. The weights were monitored weekly in whole research.

#### Implant Insertion

Femoral distal condyles were chosen as a standard site to insert implants as previously described^23^, and the implantation surgeries were performed under general anesthesia. A skin incision was made over the lateral femur-tibia joint, the femoral distal condyle was exposed after the muscle and fascia tissues were peeled away. The implant, which was 2.3 mm in diameter and 5 mm in length, was inserted into the prepared hole with an implant placement torque of 5–10 N·cm. The incision was closed with sutures in a single layer. An intramuscular injection of penicillin (200 thousand IU kg^−1^) was given to each animal once daily for 5 days postoperatively to prevent infection, and the animals were allowed unrestricted activity.

#### Impact Loading

**Figure 1:**
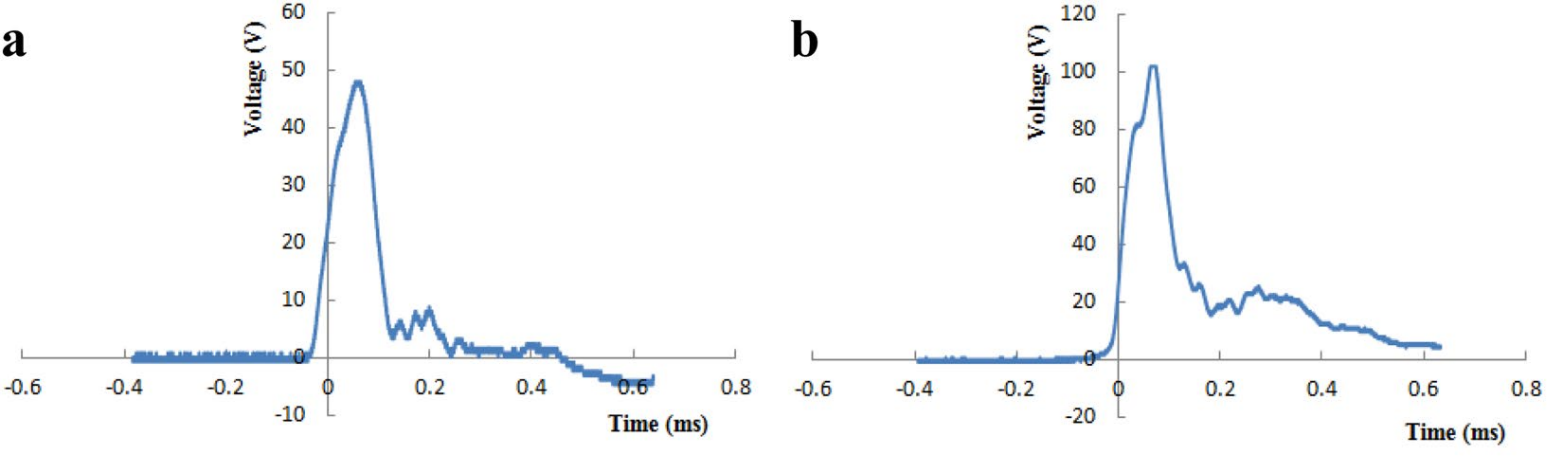
Impulse waveforms. Animals received a final impact load of 500 N (**a**) or 1000 N (**b**) in 0.2 ms.

Three months later, impact experiments were performed after favorable osseointegration occurred. The animals were randomly divided into two experimental groups suffering impact load and one control group without impact. The impact protocol^24, 25^ is illustrated in the Supplementary Fig. S1. During impact loading, a 25 g or 50 g impact mass was dropped from a height of 1 m onto a pressure sensor attached to an implant. The masses were arrested immediately to prevent multiple impacts. The voltage signals received from the pressure sensor were magnified by a charge amplifier (10 N/V), and the impulse waveforms were captured with an oscilloscope. Animals in experimental groups received a final impact load of 500 or 1000 N in 0.2 ms in order to observe the trabecular microdamage around the implant, meanwhile, prevent the detachment of the implant based on the results of our pre-experiments (Fig. 1). Then, animals were sacrificed on day 0, 7, 14, or 28, and the implants and surrounding bone were harvested for micro-CT, histomorphometry, immunofluorescence (IF) and RT-qPCR analysis. The sample sizes at each time point are illustrated in Table 1. In the experiment, one femur was dissected for micro-CT, histomorphometry and immunofluorescence randomly, and another femur from the same animal was used for RT-qPCR. So the total sample size was 60, and n (Table 1) indicates the numbers of the femurs from different rabbits. In order to investigate the characteristics of microdamage, the samples of the two experimental groups at 0d were only used for micro-CT and histomorphometry analysis. The 1000 N group was chosen for the study of peri-implant bone remodeling following impact due to its more typical microdamage. The sample size of the control group for statistical analysis was the sum of that at every time point, for the control group was not treated with an impact load.

**Table 1:** Sample Sizes at Each Time Point.

Day	Analysis	n		
		500 N	1000 N	Control
0	Micro-CT & Histology & IF/RT-qPCR	6/−	6/−	2/2
7	Micro-CT & Histology & IF/RT-qPCR	−	5/5	2/2
14	Micro-CT & Histology & IF/RT-qPCR	−	5/5	2/2
28	Micro-CT & Histology & IF/RT-qPCR	−	6/6	2/2

^n^Indicates the numbers of the femurs from different rabbits.

### Micro-computed Tomography Analysis

**Figure 2:**
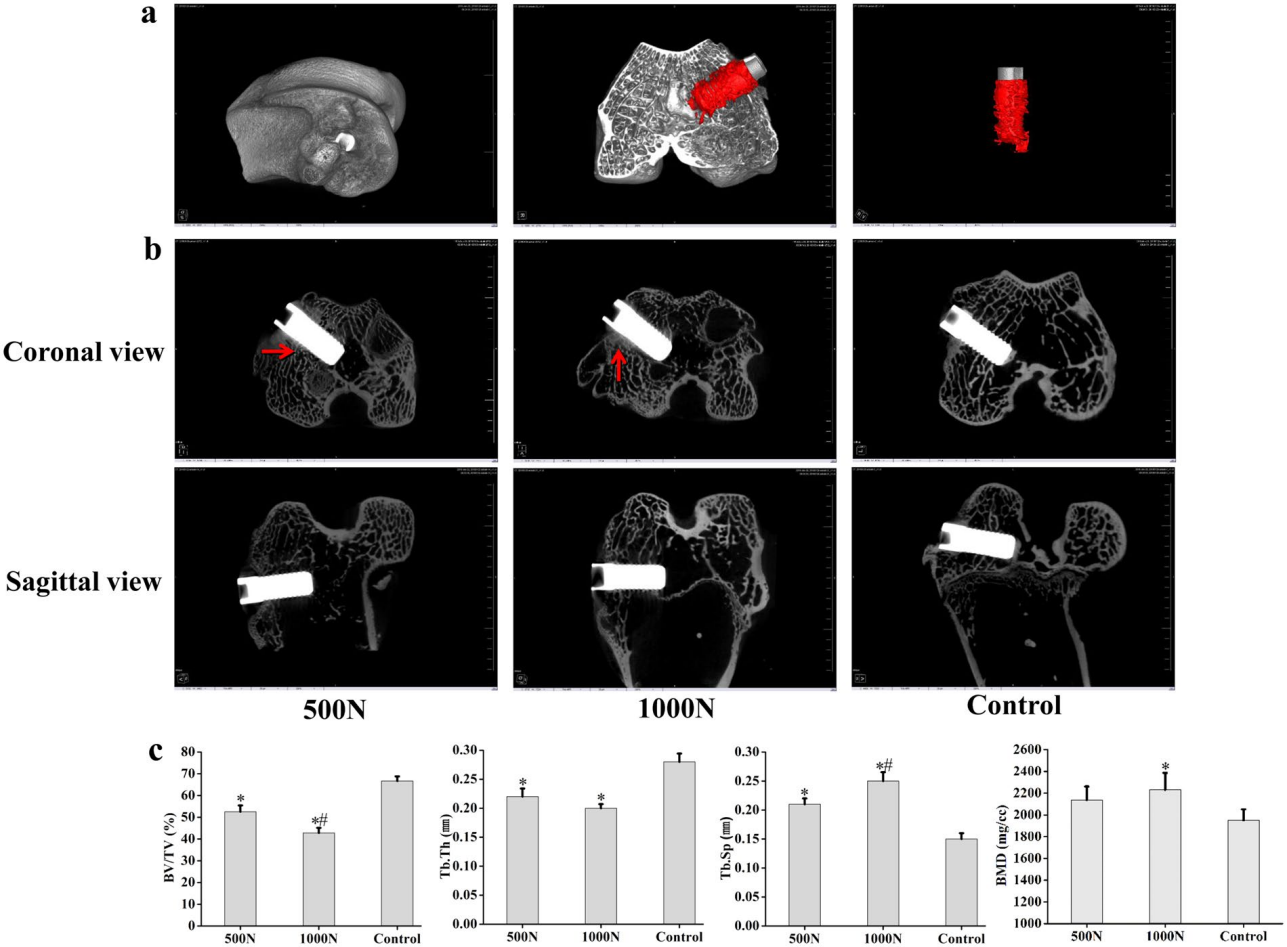
Results of Micro-CT scan and analyses. (**a**) Micro-CT images of implant and bone 3 months after implantation show a favorable osseointegration. (**b**) Damage of peri-implant bone under impact load derived from micro-CT scan. Red arrows indicate fractured trabeculae (experiment n = 6, control n = 8). (**c**) Micro-CT analysis of BV/TV, Tb.Th, Tb.Sp and BMD in the ROI. Values are expressed as means ± SD. *P < 0.05 vs. the control group and ^#^P < 0.05 vs. the 500 N group.

Bones containing implants were dissected, fixed in 4% paraformaldehyde and scanned by micro-CT (Inveon Research Workplace 2.2, Siemens Inc., Germany) at a resolution of 19.64 μm with an X-ray voltage of 80 kV and a current of 50 μA. The scanner software (Inveon Acquisition Workplace 2.2, Siemens Inc., Germany) was applied for image reconstruction and analyses. The bone around the implant with 0.5 mm thickness and 5.5 mm length (including 5 mm length of implant and 0.5 mm length of bone under the implant) was chosen as the region of interest (ROI) in each sample for microstructure analyses^26^ (Fig. 2a), and parameters such as trabecular bone volume/total volume (Tb.BV/TV, %), trabecular number (Tb.N, mm^−1^), trabecular thickness (Tb.Th, mm), trabecular separation (Tb.Sp, mm) and bone mineral density (BMD, mg/cc) were measured.

### Histomorphometry and Immunofluorescence

Undecalcified samples were prepared for subsequent histomorphometry studies. Samples were cleaned of soft tissue, dehydrated in graded alcohols and embedded in methyl methacrylate. Then, sections of 200 μm in thickness were cut longitudinally, ground to 20 μm and stained with Van Gieson (VG) and Hematoxylin-Eosin (H&E) for microscopic examination. Images were observed and captured with a light microscope (DMI6000, Leica Inc., Germany).

For sclerostin immunofluorescence, sections were washed in phosphate buffered saline (PBS) and blocked in goat serum for 10 min at room temperature. Endogenous peroxidases were quenched with 3% H_2_O_2_, and sections were incubated in rabbit sclerostin polyclonal antibody (Bioss Inc., China) for 24 h at 4 °C. After washing in PBS, sections were incubated with secondary antibody labeled with FITC. Images were captured with a confocal laser scanning microscope (CLSM) (Fluo View FV-1000, Olympus Inc., Japan). Fluorescence intensity was measured in five areas of each section using the CLSM software, and the average was calculated for statistical analyses.

### RNA Extraction and RT-qPCR

Bone tissue at the base of the implant was snap frozen and crushed in liquid N_2_. According to the manufacturer’s instructions, total RNA was extracted using Trizol reagent and was dissolved in DEPC H_2_O. cDNA was synthesized using a 10 μl reverse transcription reaction mixture composed of 0.5 μg total RNA, 2 μl 5× PrimeScriptTM Buffer, 0.5 μl PrimeScriptTM RT Enzyme Mix I and 0.5 μl random primer. Partial sequences of SOST, β-catenin, RANKL and β-actin in the reverse transcribed cDNA were amplified using a fluorescence RT-qPCR instrument (RG-3000, Gene Inc., Australia). The forward and reverse primer sequences used for amplification are listed in Supplementary Table S2. Relative mRNA expression levels were standardized to β-actin expression for quantified analyses using the ∆Ct method.

### Ethics

This study was carried out in strict accordance with the recommendations in the Guide for the Care and Use of Laboratory Animals of the Animal Center of the Fourth Military Medical University. The protocol was approved by the Laboratory Animal Care & Welfare Committee, Fourth Military Medical University (No: 20150201). All surgeries were performed under sodium pentobarbital anesthesia, and all efforts were made to minimize suffering.

### Statistical Analysis

The data were presented as mean ± standard deviation and analyzed using SPSS19.0 (IBM Inc., USA) for one-way ANOVA statistical analyses. Statistical significance was considered as p < 0.05.

## Results

### Bone-implant Osseointegration

Implants inserted into the rabbit femoral distal condyle were stable by 3 months after implantation. Micro-CT scan was performed to study the osseointegration at this time. The image showed that the areas around the implant were full of trabeculae. The intact and thick trabeculae were arranged regularly and distributed around and at the bottom of the implant. A good interface was observed between the implant and the bone (Fig. 2a).

### The Microdamage of Bone around the Implant and the Failure of the Implant-bone Interface

#### Histological Study

**Figure 3:**
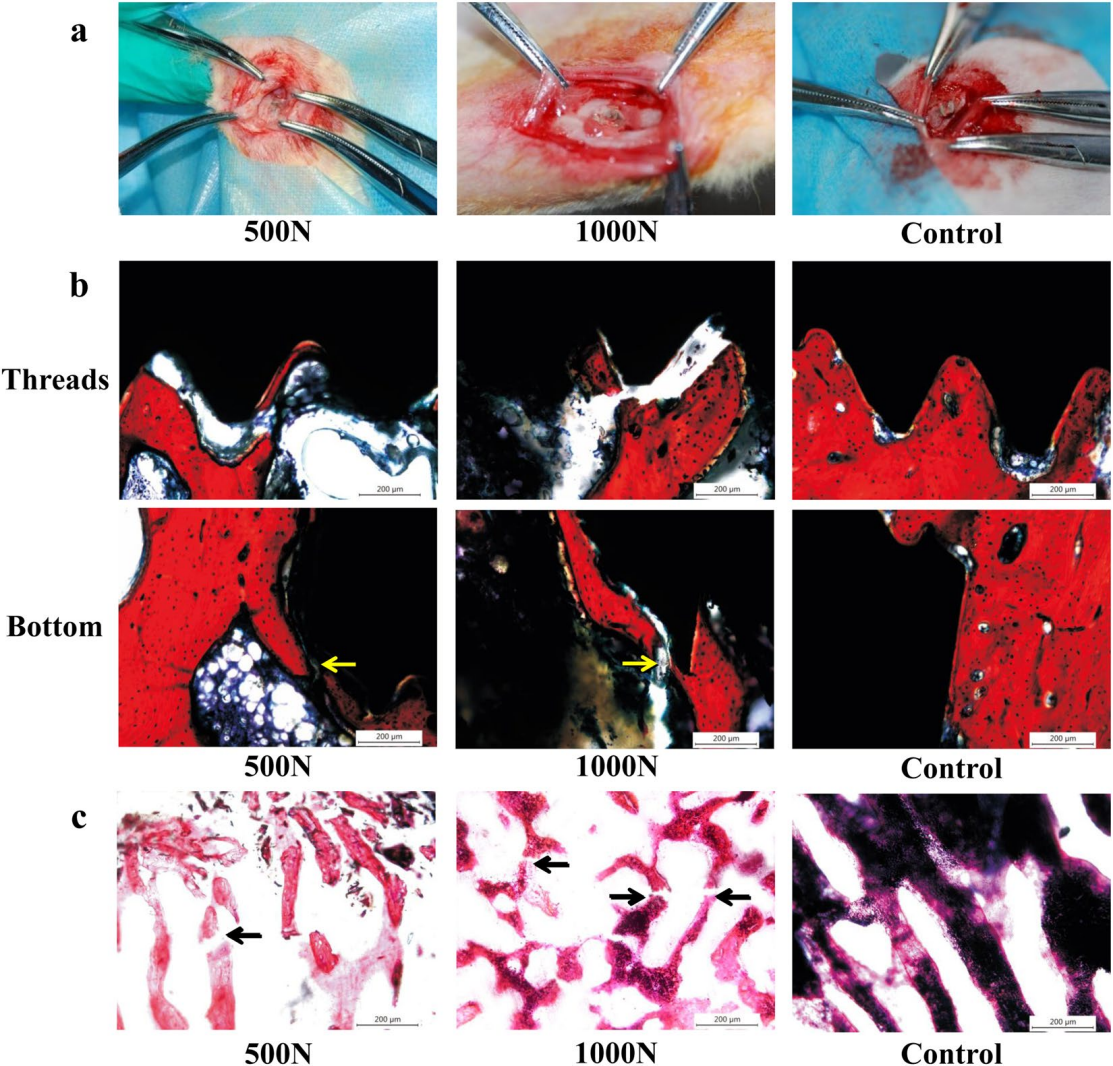
The results of bone damage under impact. (**a**) Morphology of the cortical bone around the implant under impact load. (**b**) Damage to the osseointegration among the threads and at the bottom of implant with VG staining (10×). Yellow arrows show trabeculae fracture at the bottom of implant. (**c**) Histomorphometry of impacted peri-implant bone with H&E staining (10×). Black arrows indicate trabeculae fracture.

No obvious change in the cortical bone around the implant was observed after impact (Fig. 3a). However, histological study showed that the osseointegration failed at the interface between the bone and the implant. Fractured trabeculae were observed around and at the bottom of the implant.

The implant and bone stained with VG are shown in Fig. 3b. In this figure, new bone appeared around the implant threads and at the bottom of implant by 3 months after insertion, and trabeculae were regularly arranged in the control group, which indicated that the osseointegration was favorable. After impact loading, bone tissues in some areas were broken away from the implant, and the trabeculae at the bottom of implant were fractured. This demonstrated that the interface between the implant and the surrounding tissues was incomplete in the experimental groups.

Similar microdamage in peri-implant bone is shown in Fig. 3c with H&E staining. Trabeculae around the implant were fractured and disordered under impact loading, while trabeculae in the control group were regularly arranged. Damage photography of interface and surrounding bone was more severe in group 1000 N than in group 500 N.

#### Micro-CT Analyses

The image of micro-CT shows similar failed osseointegration and fractured trabeculae around and at the bottom of the implant (Fig. 2b). To describe the microdamage quantitatively, micro-CT analyses were performed in this study. The results demonstrated that BV/TV and Tb.Th decreased, while Tb.Sp and BMD increased compared with the control group (p < 0.05). The results indicated that the bone mass decreased after impact damage, but BMD increased when the trabeculae were fractured and compressed. There were statistically significant differences in BV/TV and Tb.Sp between the test groups (p < 0.05), which indicated that the damages were different under impact load (Fig. 2c).

### The Peri-implant Bone Healing Process: From Impact Damage to Remodeling

**Figure 4:**
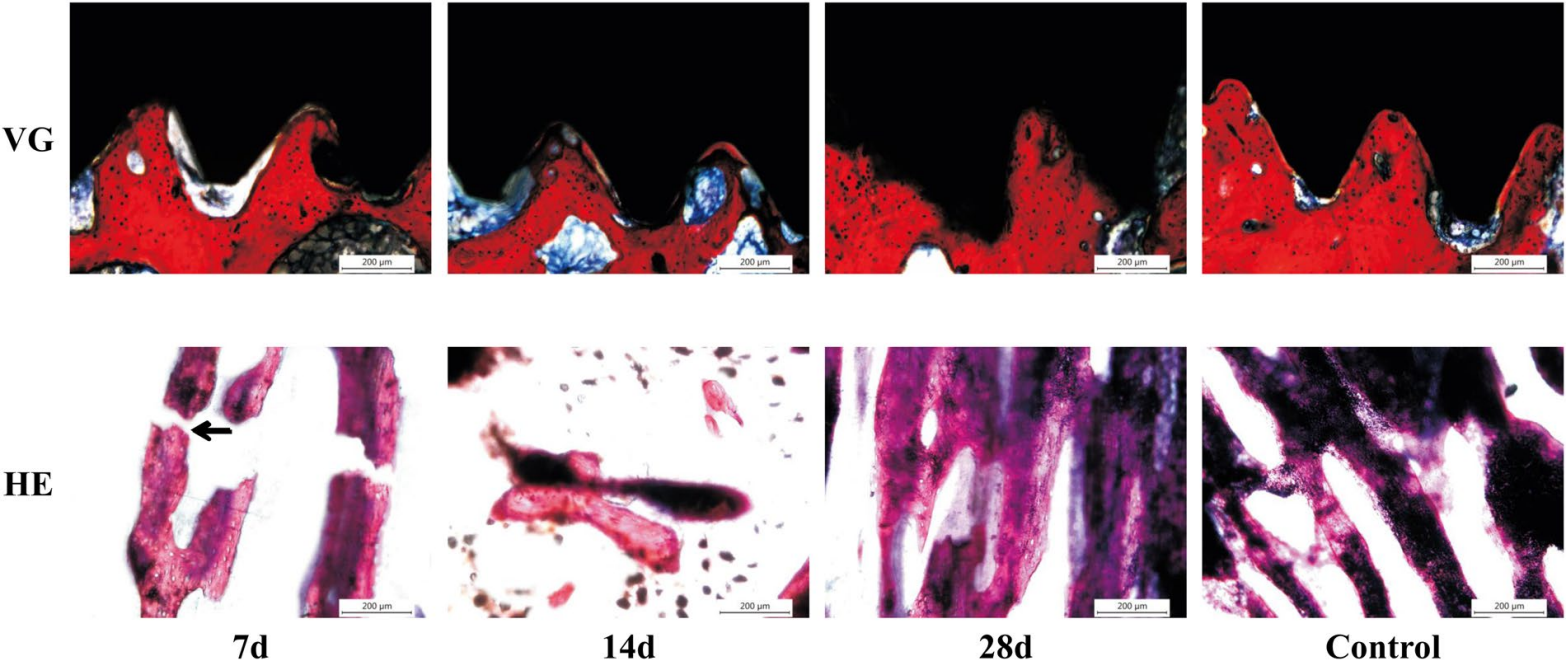
Histomorphometry of the osseointegration among the threads and peri-implant bone in the remodeling process following impact injury with VG and H&E staining (10×). Black arrow indicates trabeculae fracture.

To investigate the remodeling of peri-implant bone after impact damage, bone structures along the interface between the implant and the bone were studied at different time points. Cancellous bones around the implant are shown in Fig. 4.

At day 7 after impact, the gap between the implant and the bone was observed in VG stained sections and the structure of the trabeculae stained with H&E was broken (Fig. 4). The gaps attributed to impact damage were extended, and the trabeculae were scattered at day 14, which indicated that bone absorption may have occurred in this area. By the last time point, the gaps disappeared, and regularly arranged trabeculae were observed. There was no significant difference in the microstructure compared with the control group, which demonstrated that the osseointegration had completely reformed by day 28.

**Figure 5:**
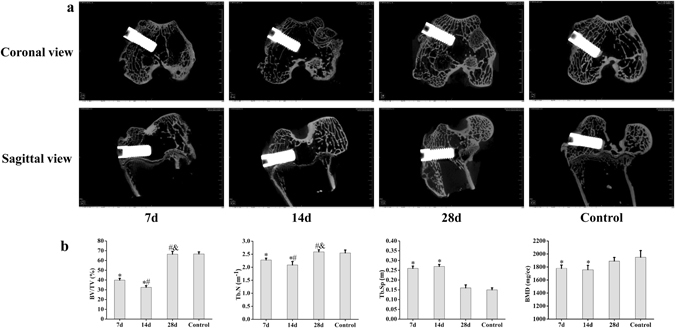
Micro-CT scan and analyses of peri-implant bone during remodeling after impact. (**a**) Remodeling of peri-implant bone at different time points after impact derived from micro-CT scan. (**b**) Micro-CT analysis of BV/TV, Tb.N, Tb.Sp and BMD in the ROI at different time points (7d and 14d n = 5, 28d n = 6, control n = 8). Values are expressed as means ± SD. *P < 0.05 vs. the control group, ^#^P < 0.05 vs. 7d, and ^&^P < 0.05 vs. 14d.

Micro-CT scanning and analyses were applied to quantify the changes in trabecular microstructure (Fig. 5). It could be observed that BV/TV, Tb.N decreased while Tb.Sp increased 14 days after impact (p < 0.05). And BMD decreased at day 7 and 14 after temporary increasing at day 0. There was no significant difference in these parameters between the experimental and control group by day 28. These results were consistent with the histomorphological results.

### Immunofluorescence Staining of Sclerostin in Trabeculae after Impact

**Figure 6:**
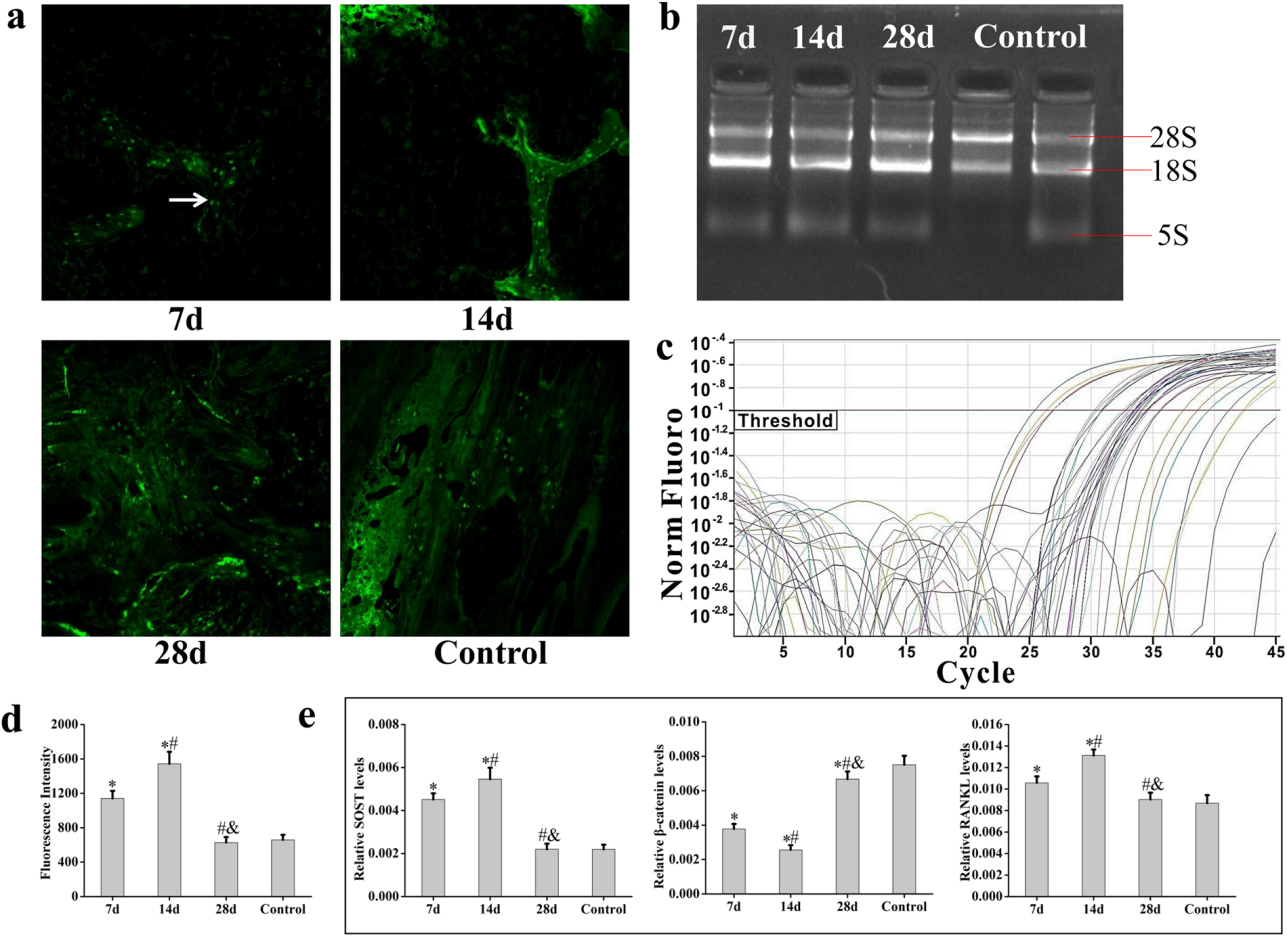
The expression of sclerostin, β-catenin and RANKL of peri-implant bone during remodeling after impact. (**a**) Immunofluorescence staining on sclerostin in trabeculae at different time points after impact. White arrow indicates the expression of sclerostin. (**b**) Extracted total RNA in agarose gel electrophoresis. In this figure, 3 bands representing 28 s, 18 s and 5 s rRNA are showed clearly, which demonstrates that the extracted total RNA was not degraded. (**c**) The SOST PCR amplification curve. The marker in this figure represents the threshold of SOST in PCR amplification. (**d**) The fluorescence intensity of sclerostin immunofluorescence staining. (**e**) The expression of SOST, β-catenin and RANKL mRNA (7d and 14d n = 5, 28d n = 6, control n = 8). Values are expressed as means ± SD. *P < 0.05 vs. the control group, ^#^P < 0.05 vs. 7d, and ^&^P < 0.05 vs. 14d.

Immunofluorescence staining was used to study the expression of sclerostin during the process of remodeling following bone impact damage. Immunofluorescence images are shown in Fig. 6a. In this figure, the sclerostin protein staining is indicated by the green fluorescence points, and sclerostin protein expression is measured as fluorescence intensity in Fig. 6d. From the results (Fig. 6d), it can be observed that the expression of sclerostin was higher than that in the control group on days 7 and 14 after impact (p < 0.05). During the process of remodeling after damage, it increased significantly from day 7 to 14 (p < 0.05). However, there was no obvious difference in expression of sclerostin between the experimental and control group by day 28.

### The Expression of SOST, β-catenin and RANKL mRNA in Bone Tissue around the Implant after Impact

RT-qPCR was used to quantify the expression of SOST and to correlate sclerostin expression with β-catenin and RANKL. Figure 6b shows the extracted total RNA in agarose gel electrophoresis and Fig. 6c illustrates the SOST PCR amplification curve. The expression of SOST, β-catenin and RANKL mRNA was quantified using the value of 2^−∆Ct^ in Fig. 6e. In the figure, the expression of SOST and RANKL mRNA increased rapidly after impact compared with the control group, and the values reached a maximum at day 14 (p < 0.05). Then, the expression of SOST and RANKL mRNA decreased gradually to the control group levels at day 28. The expression of SOST mRNA was similar to sclerostin protein expression based on the immunofluorescence staining results. The expression of β-catenin mRNA was opposite to that of SOST and RANKL mRNA, and it did not return to the level of the control group completely until day 28 (p < 0.05).

## Discussion

When an implant denture experiences an impact load, there will be microdamages in the peri-implant bone and failure of the implant-bone interface. In addition, the expression of proteins will be changed to modify the bone structure correspondingly. In this study, implants were inserted into the femoral distal condyles of New Zealand white rabbit, and an impact load was applied to the implants after osseointegration occurred. The features of bone damage around the implant and the remodeling of bone were studied through micro-CT analyses and hard tissue slicing with VG and H&E staining. Further, the expression of sclerostin, β-catenin and RANKL were analyzed by immunofluorescence and RT-qPCR during the process of remodeling following bone damage. The results showed that there was no significant change in the cortical bone around the implant, but debonding at the interface and impaired osseointegration in specific areas around the implant were observed. Microdamage in cancellous bone was also observed around the implant. The expression of sclerostin, β-catenin and RANKL correlated with the bone damage and process of remodeling. These data indicate that sclerostin may be involved in bone formation and resorption caused by impact through regulating the Wnt/β-catenin and RANKL/RANK pathways. The results of this study reveal the characteristics of the impact damage to the bone around the implant, and provide a reference for damage assessment and clinical treatment of patients with impact loading.

Impact load is a transient load that transmits through or reflects between an implant and bone in stress waves when the pulse duration of the load is in the microsecond range. The stress waves not only spread through the implant-bone interface but also reflect at the interface when they pass through the anisotropic composite structure of the implant and bone^9, 27^. The impact energy is spread and dissipated quickly along the interface, which may cause the failure of the implant-bone interface and surrounding bone when the energy cannot be absorbed and buffered by this composite structure. However, the characteristics of impact damage in the bone around the implant have not been reported. In this study, microdamage of peri-implant bone under impact was investigated. The results showed that the bone tissue was broken away from the implant and fractured trabeculae around the implant were observed in histology, although no obvious change was found in the cortical bone around the implant. Micro-CT analyses demonstrated that trabecular thickness (Tb.Th) decreased while trabecular space (Tb.Sp) increased after the cancellous bone was fractured and exposed to the impact compression. This change of trabecular structure also led to the decrease of bone volume (BV) and its percentage in total volume (BV/TV). Meanwhile, BMD increased accordingly. And the damages correlated significantly with the impact energy. The results denote that the failure of the interface and cancellous bone may be attributed to the transmission of stress waves and reveal the characteristics of bone damage caused by impact in this composite structure. Together, these results suggest that injuries at the implant-bone interface and in the cancellous bone around the implant are invisible but should be given more attention to evaluate the condition of the impact damage and provide guidelines for clinical treatment.

To clinically maintain implants after they sustain an impact load, it is also important to study the processes of bone remodeling and osseointegration formation. In the micro-CT analysis, trabecular number (Tb.N) is defined as the number of trabecular intersections between bone and other tissues in the ROI. Thus, it is usually used to describe changes in trabeculae during bone remodeling. In this study, the trabeculae were first found fractured after impact, which would lead to an increase in the number of intersections and the corresponding Tb.N. However, Tb.N decreased with the increase of Tb.Sp in this study. The main reason for this may be that bone loss occurred around the implant, and this phenomenon could be indicated by a reduced BV/TV. The change of BMD also reflected the structure alteration of trabeculae, and the value of BMD turned to decrease at day 7 and 14 after temporary increase at day 0. All these results demonstrate that bone remodeling may be present as resorption 14 days after impact. The process of remodeling was also confirmed by the histological results that the implant-bone interface was damaged and marrow was found to grow into the gap between the implant and the bone. From day 14 to 28, the bone mass and BMD surrounding the implant increased, with increases in both Tb.N and BV/TV, and intact and regularly arranged trabeculae were gradually observed histologically. By day 28, there was no significant difference in the bone mass and BMD compared with the control group. The microstructure of the peri-implant bone returned to normal morphology, and a favorable osseointegration reformed. These results indicate that bone formation is dominated in the process of bone remodeling from day 14 to 28. From these results, it could be observed that the characteristics of the impact damage of bone around the implant were different than that of clinically normal surgical insertion, but the processes of bone remodeling were similar for the formation of the osseointegration around the implant. Therefore, the principles for treatment after insertion surgery could be used as a reference for the management of impact-loaded patients.

Sclerostin encoded by the SOST gene is a glycoprotein secreted by osteocytes^28–30^. Previous studies have reported that there are alterations in its expression in animal models of bone defects or fractures^31–34^. Changes in the microstructure could cause fluid flow in the bone matrix, which would change the mechanical environment surrounding the osteocytes^11^. This mechanical signal is sensed and conducted by osteocytes though the lacunar-canalicular system^15, 35, 36^, and then sclerostin is secreted by osteocytes to adapt to the biomechanical environment^37–39^. In this study, the expression of sclerostin continued to increase after impact and reached a maximum at day 14. Then, it decreased gradually to normal levels at day 28. From the results above, it can be observed that the expression of sclerostin is related with the process of bone damage and remodeling.

**Figure 7:**
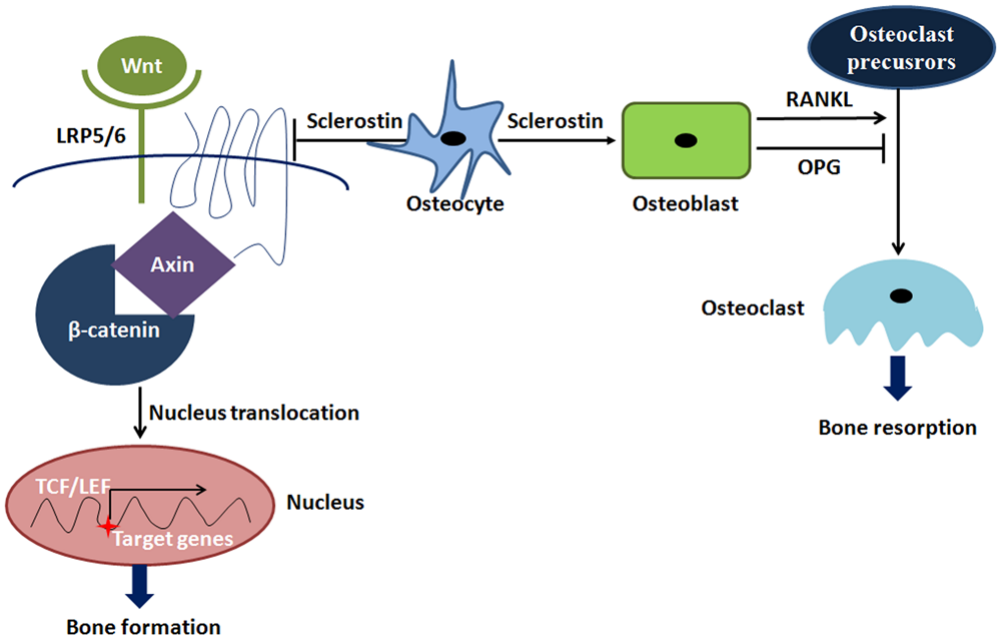
The relationship of sclerostin with Wnt/β-catenin and RANKL/RANK.

Other studies have reported that sclerostin is an effective antagonist of bone formation^40–42^. The nodular cystine domain in sclerostin allows the protein to competitively bind to the Wnt co-receptor LRP5/6 of the osteoblast. This reduces β-catenin nuclear translocation. As a result, osteoblast activity is decreased, and new bone formation and mineralization are inhibited^43, 44^. Meanwhile, sclerostin could promote the secretion of RANKL from osteoblasts, which would stimulate the differentiation of osteoclasts from precursors and accelerate bone resorption^22^. The relationship of sclerostin with the Wnt/β-catenin and RANKL/RANK pathways is illustrated in Fig. 7.

In this study, the expression of RANKL mRNA continued to increase with the increase of SOST, while β-catenin decreased over the first 14 days after impact. Then, the expression of RANKL and SOST mRNA decreased gradually to the level of the control group, while, in contrast, β-catenin increased by day 28. The aforementioned changes in protein expression and mRNA levels were consistent with the behavior of peri-implant bone absorption and formation in the micro-CT and histological results. These results suggest that sclerostin may be involved in both bone anabolism and catabolism in response to mechanical stimulation by regulating the Wnt/β-catenin and RANKL/RANK pathways, respectively. The lower expression of β-catenin than that of control group at day 28 in this study indicated that it didn’t reach the normal value. The main reason might be that it will take longer for the cancellous bone to return to normal completely, and β-catenin may continue to increase during this process.

In future experiments, the expression of β-catenin and RANKL should be investigated when the expression of sclerostin is down-regulated, and the change in bone microstructure should also be analyzed to determine that whether sclerostin serves as a potential target to regulate bone remodeling under damage through Wnt/β-catenin and RANKL/RANK pathways, which would provide a new therapeutic target for patients with dental implant to improve the osseointegration by regulating the expression of sclerostin.

## Electronic supplementary material

Supplementary Information
