# Supplementary material for: The Microdamage and Expression of Sclerostin in Peri-implant Bone under One-time Shock Force Generated by Impact
Source: Sci Rep. 2017 Jul 26;7:6508. doi: 10.1038/s41598-017-06867-9 (PMC5529451; doi:10.1038/s41598-017-06867-9)
Supplement: Supplementary file 1 — Supplementary Information [file 41598_2017_6867_MOESM1_ESM.pdf]

The Microdamage and Expression of Sclerostin in Peri-implant Bone under One-time Shock Force Generated by Impact

Xiaoou Diao[1,2,3], Zhirui Li[1,2,3], Baili An[1,2,3], Haitao Xin[1,2,3,*], Yulu Wu[1,2,3], Kai Li[1,2,3], Fan Feng[1,2,3], Chenyun Dou[1,2,3]

[1] State Key Laboratory of Military Stomatology, Department of Prosthodontics, School of Stomatology, The Fourth Military Medical University, Xi'an 710032, China

[2] National Clinical Research Center for Oral Diseases, Department of Prosthodontics, School of Stomatology, The Fourth Military Medical University, Xi'an 710032, China

[3] Shannxi Key Laboratory of Stomatology, Department of Prosthodontics, School of Stomatology, The Fourth Military Medical University, Xi'an 710032, China

* Corresponding author

E-mail address: xhthmj@fmmu.edu.cn.

Tel./fax: + 86 029 84776464.

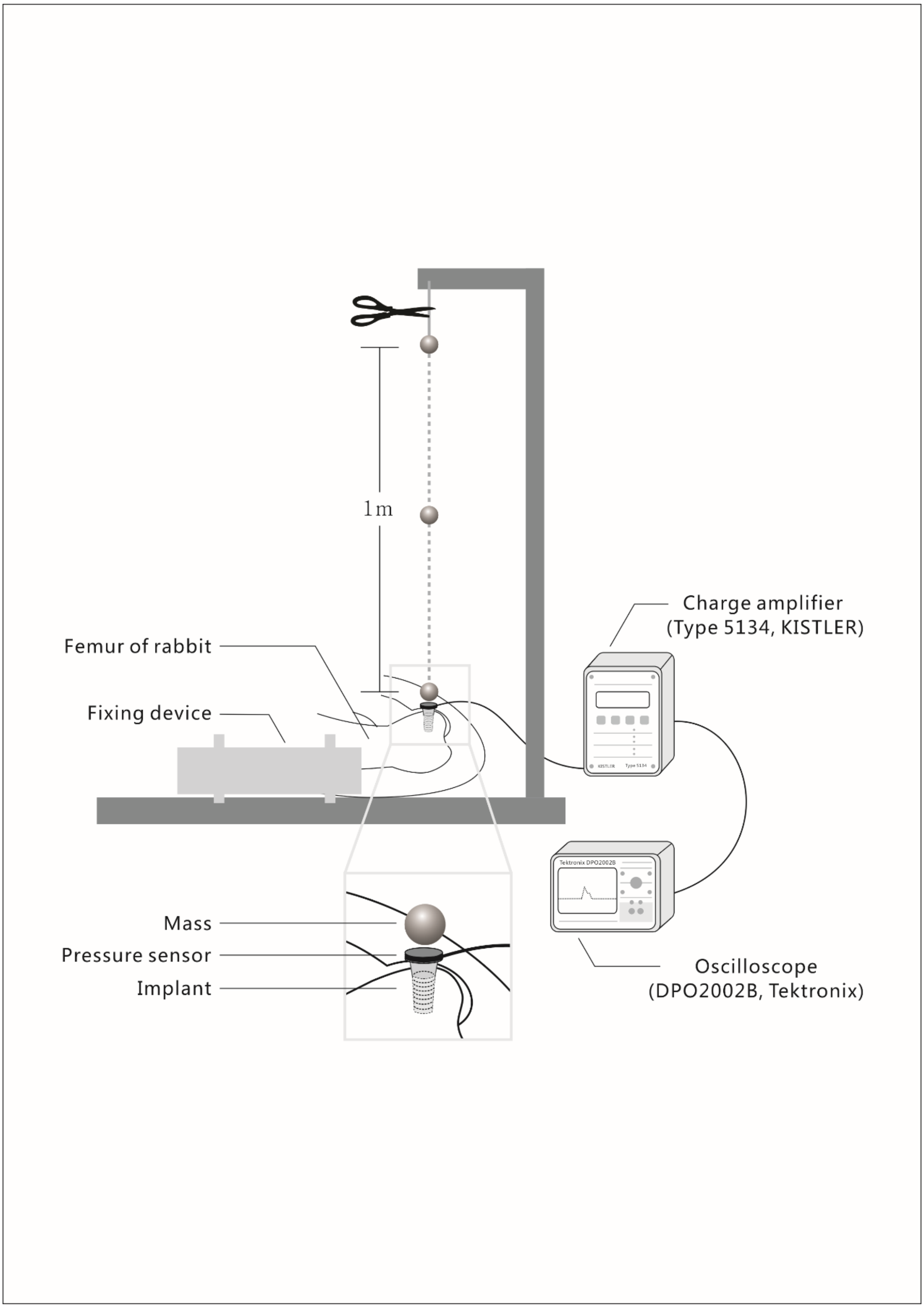

Supplementary Fig. S1. The illustration of impact. During impact loading, an impact mass is dropped from a height of 1 m onto a pressure sensor attached to an implant when the thread is cut off. The voltage signal received from the pressure sensor is magnified by a charge amplifier (10N/V), and the impulse waveform is captured with an oscilloscope.

Supplementary Table S2

The Forward and Reverse Primer Sequences for Amplification

	Forward Primers	Reverse Primers
SOST	TGGTCCTGACTCTGCCACTTG	GCCTCTGTTTCTGTCTCCCTCTC
β-catenin	CATCTGTGCTCTCCGTCATCTGA	CAACTGAACTAGGCGTGGAATGG
RANKL	ATGTCCTCCTGGCACCTACCT	CCCTTCCTCGCATTCACACAC
β-actin	CACCCTCTCTCTCGACGAAACC	CGGCCACATTGCAGAACTTTG
